# Supplementary material for: Overview of Antifungal Drugs against Paracoccidioidomycosis: How Do We Start, Where Are We, and Where Are We Going?
Source: J Fungi (Basel). 2020 Nov 19;6(4):300. doi: 10.3390/jof6040300 (PMC7712482; doi:10.3390/jof6040300)
Supplement: Supplementary file 1 [file jof-06-00300-s001.pdf]

SUPPLEMENTARY MATERIAL

Overview of antifungal drugs against Paracoccidioidomycosis: How do we start, where are we and where are we going?

Lívia do Carmo Silva[1,2], Amanda Alves de Oliveira[1,2], Dienny Rodrigues de Souza[1,2], Katherine Lohany Barros Barbosa[1,2], Kleber Santiago Freitas e Silva[1], Marcos Antonio Batista de Carvalho Júnior[1], Olívia Basso Rocha[1], Raisa Melo Lima[1,2], Thaynara Gonzaga Santos[1,2], Célia Maria de Almeida Soares[1], Maristela Pereira*[1]

[1] Institute of Tropical Pathology and Public Health, Federal University of Goiás, Goiânia, Goiás, Brazil.

[2] Institute of Biological Sciences, Laboratory of Molecular Biology, Federal University of Goiás, Goiânia, Goiás, Brazil.

Supplementary Table 1. Antifungal derived from synthetic and semi-synthetic compounds tested against *Paracoccidioides* spp.

Compounds	Target/Modes of action	MIC	MFC	Ref.
Amphotericin B	Cell membrane	0.0078-1.0 µg/mL	0.002-0.5 µg/mL	[1–5]
Fluconazole	Ergosterol synthesis	0.062-0.125 µg/mL	ND	[1,2]
Itraconazole	Ergosterol synthesis	0.002-0.5 µg/mL	0.002-0.5 µg/mL	[1–3,6]
Ketoconazole	Ergosterol synthesis	0.0009-0.015 µg/mL	ND	[1,2]
Isavuconazole	Ergosterol synthesis	0.001 µg/mL	ND	[6]
Saperconazole	Ergosterol synthesis	$6.7x10^{-2}$-$6.7x10^{-5}$ µg/mL	$6.7x10^{-2}$-$6.7x10^{-5}$ µg/mL	[7]
Thiazole derivatives (1-7, 9 and 12)	ND	62.5-250 µM	ND	[8]
Caspofungin	Synthesis of 1,3-β-d-glucan	ND	ND	[9]
Micafungin	Synthesis of 1,3-β-d-glucan	4-64 µg/mL	ND	[10]
Sulfametoxazole	Folate biosynthesis	9.37-150 µg/mL	ND	[2]
Sulfadiazine	ND	12.5-200 µg/mL	ND	[11]
Sulfadimethoxyne	ND	12.5-200 µg/mL	ND	[11]
Sulfametoxazole-trimethoprim	Folate biosynthesis	1.6-300 µg/mL	ND	[2,12]
Terbinafine	Ergosterol synthesis	0.0078-1.0 µg/mL	ND	[2,6]
Lapachol	Cell membrane	0.13-0.26 µM	ND	[13]
Semicarbazone derivatives of lapachol	Cell membrane	0.42-1.03 µM	ND	[13]
Thiosemicarbazone lapachol	Cell membrane	0.01-0.1 µM	ND	[13]
Thiosemicarbazone derivatives of lapachol	Cell membrane	3.90-62.5 µg/mL	3.9-62.5 µg/mL	[14]
Thiosemicarbazide	Promotes oxidative stress	344 µM	ND	[15]
Camphene derivative of Thiosemicarbazide	Promotes oxidative stress	79 µM	ND	[16]
6-quinolinyl/Chalcones (3b and 3e)	Cell membrane	1.95-31.25 µg/mL	1.95-31.25 µg/mL	[17]
6-quinolinyl/Chalcones (3a-g)	Cell membrane	7.8-500 µg/mL	ND	[18]
6-quinolinyl N-oxide/Chalcones (4a-g)	Cell membrane	1.90-500 µg/mL	ND	[18,19]

Chalcones (5a-g)	Cell membrane	31.20-500 µg/mL	ND	[18]
Quinolinyl N-oxide/ Chalcones (1-8)	Cell membrane	2.9-42.2 µM	5.8-104.3 µM	[20]
Ferrostatin-1	ND	1.3 µM	ND	[21]
Gallic acid (G1)	N-Glycosylation	4-31 µg/mL	ND	[22]
Alkyl gallates (G2-G17)	N-Glycosylation	0.004-16 µg/mL	ND	[22,23]
Ruthenium dithiocarbamate compounds (C1-C9)	ND	4-128 µg/mL	ND	[24]
Cilofungin (LY121019)	Cell wall biosynthesis	10-20 µg/mL	ND	[25]
Phenethylamine complexed with diphenylphosphine	Apoptosis- and autophagy-like mechanisms	0.01-0.76 µg/mL	ND	[26]
CP1 compound	Chorismate synthase	2-32 mg/L	4-128 mg/L	[27]
CS8	Chorismate synthase	512 µg/mL	512 µg/mL	[28]
CaCS02	Chorismate synthase	32 µg/mL	32 µg/mL	[28]
ZINC4559339	Isocitrate lyase	7.3-15.6 µg/mL	7.3-15.6 µg/mL	[29]
Reduced argentilactone	Isocitrate lyase	30.2-85 µM	ND	[30]
Miltefosine	Thioredoxin reductase	0.12-1.0 µg/mL	ND	[31]
Oxadiazol compounds (LMM5 and LMM11)	Thioredoxin reductase	1-32 µg/mL	1-32 µg/mL	[32]
F0876-0030	Thioredoxin reductase	32 µg/mL	77.4-309.5 µM	[33]
F1109-0100	Thioredoxin reductase	128 µg/mL	ND	[33]
F1806-0122	Thioredoxin reductase	8-16 µg/mL	38.7-309.5 µM	[33]
F3010-0057	Thioredoxin reductase	64 µg/mL	ND	[33]
F3222-4930	Thioredoxin reductase	64-128 µg/mL	ND	[33]
F3307-0033	Thioredoxin reductase	128 µg/mL	ND	[33]
F3307-0100	Thioredoxin reductase	8-16 µg/mL	16.9-134.9 µM	[33]
F3394-0412	Thioredoxin reductase	256 µg/mL	ND	[33]
F5754-0452	Thioredoxin reductase	64-128 µg/mL	ND	[33]
Raltegravir	Thioredoxin reductase	16-32 µg/mL	ND	[34]
Venetoclax (ABT-199)	Thioredoxin reductase	16-64 µg/mL	ND	[34]
SR9243	Thioredoxin reductase	64 µg/mL	ND	[34]
Zafirlukast	Thioredoxin reductase	16-32 µg/mL	ND	[34]
Gliquidone	Thioredoxin reductase	32-64 µg/mL	ND	[34]

Glyburide	Thioredoxin reductase	64 µg/mL	ND	[34]
HS1	Homoserine dehydrogenase	32-64 µg/mL	ND	[35]
HS2	Homoserine dehydrogenase	32-64 µg/mL	ND	[35]
HS6 (Zinc20611644)	Homoserine dehydrogenase	128 µg/mL	128 µg/mL	[36]
HS7 (Zinc15967722)	Homoserine dehydrogenase	32 µg/mL	32 µg/mL	[36]
HS9 (Zinc2123137)	Homoserine dehydrogenase	8 µg/mL	8 µg/mL	[36]
4-methoxy-naphtalene derivative	Homoserine dehydrogenase	8-128 µg/mL	ND	[37]
4-methoxynaphthalene-N-acylhydrazones (4a-c, 4g and 4k)	ND	0.5-64 µg/mL	ND	[38]
Azasterol analogs (AZA-1, AZA-2 and AZA-3)	Sterol biosynthesis	0.5-10 µM	ND	[39]
Hydrazone derivatives (9, 10, 11 and 12)	Sterol biosynthesis	0.1-5 µM	ND	[40]
Alkaloid compounds (3, 4, 6 and 8)	Malate synthase	4.04-249.57 µg/mL	ND	[41]
Peptide of the mastoparan class (MK58911)	ND	7.8-15.6 µg/mL	ND	[42]
Peptide derived from lactoferrin	ND	0.63-1.25 µg/mL	ND	[43]
Rifampicin	RNA polymerase	40-80 µg/mL	ND	[4]
Calix[n]arenes	ND	16-256 µg/mL	16-256 µg/mL	[44]
Vistusertib	Phosphatidylinositol 3-kinase TOR2	1.0-4.2 µM	1.0-4.2 µM	[45]
BGT-226	Phosphatidylinositol 3-kinase TOR2	3.6-7.3 µM	7.3-14.5 µM	[5]
Dactolisib	Phosphatidylinositol 3-kinase TOR2	8.3-66.6 µM	33.2-266.2 µM	[5]
Dexlansoprazole	Na+/K+-exchanging ATPase alpha chain	21.1-169.2 µM	84.6-338.4 µM	[5]
Albendazole	Tubulin beta chain	58.9-29.4 µM	58.9-235.6 µM	[5]
Mebendazole	Tubulin beta chain	3.3-26.4 µM	13.2-26.4 µM	[5]
Bifonazole	Lanosterol 14-alpha demethylase	0.2-0.8 µM	0.2-0.8 µM	[5]
Sertaconazole	Lanosterol 14-alpha demethylase	0.0036 µM	0.0150 µM	[5]
Butoconazole	Lanosterol 14-alpha demethylase	0.001-0.002 µM	0.001-0.002 µM	[5]

Luliconazole	Lanosterol 14-alpha demethylase	0.0005-0.0007 µM	0.0013-0.0026 µM	[5]
Midostaurin	Protein kinase C	13.7-54.7 µM	27.3-219.0 µM	[5]
Raltitrexed	Thymidylate synthase	68.0 µM	272.6 µM	[5]
ENMD-2076	Serine/threonine-protein kinase	7.4-14.8 µM	7.4-14.8 µM	[5]
Tozasertib	Serine/threonine-protein kinase	16.8-269.1 µM	33.6- 269.01 µM	[5]

MIC – minimal inhibitory concentration, MFC – minimum fungicidal concentration, ND – not determined.

Supplementary Table 2. Antifungal compounds derived from plants and microorganisms' sources tested against *Paracoccidioides* spp.

Source	Compounds/Extracts	MIC	MFC	Ref.
**Derivated Plants**				
Hyptis ovalifolia	Argentilactone	4.5-36 μg/mL	4.5-36 μg/mL	[1]
Eugenia uniflora	Oenothein B	62.5-500 μg/mL	125-500 μg/mL	[46,47]
Schinus terebinthifolius	schinol	7.5-125 μg/mL	7.5-125 μg/mL	[48]
	Bifenil: 4'-etil-4-metil-2,2', 6,6' -tetra-hidroxi [1,1'-bifenil] -4,4'-dicarboxilato)	15.6-250 μg/mL	15.6-250 μg/mL	[48]
Blepharocalyx salicifolius	Essential oil	156.25 μg/mL	ND	[49]
Artocarpus heterophyllus	ArtinM	ND	ND	[50]
Allium sativum	Ajoene	0.05-0.0125 mg/mL	ND	[51]
Schinus molle	Essential oil	39.06 μg/mL	ND	[52]
Glycine max	Fatty acid methyl esters	15.6-125 μg/mL	ND	[53]
Zea mays	Fatty acid methyl esters	62.5-500 μg/mL	ND	[53]
Helianthus annuus	Fatty acid methyl esters	31.2-250 μg/mL	ND	[53]
Punica granatum	Hexane, EtOH, AcOEt, DCM, Aquous	40-1000 μg/mL	ND	[54]
Schinus terebinthifolius	Hexane, AcOEt, DCM, Aquous	15.2-1000 μg/mL	ND	[54]
Rubus urticaefolius	Hexane, EtOH, AcOEt, DCM, Aquous	30-1000 μg/mL	ND	[54]
Piper regnellii	Hexane, EtOH, AcOEt, DCM, Aquous	7.8-1000 μg/mL	ND	[54]
Piper abutiloides	Hexane, EtOH, AcOEt, DCM	15-1000 μg/mL	ND	[54]
Inga spp.	Hexane, EtOH, AcOEt, DCM, Aquous	125-1000 μg/mL	ND	[54]
Herissantia crispa	Hexane, EtOH, AcOEt, DCM, Aquous	30-1000 μg/mL	ND	[54]
Rumex acetosa	EtOH	60-500 μg/mL	ND	[54]
Alternanthera brasiliana	EtOH	1000 μg/mL	ND	[54]
Baccharis dracunculifolia	Hexane, EtOH, AcOEt, DCM, Aquous	7.8-1000 μg/mL	ND	[54]
Baccharis dracunculifolia	Caryophyllene oxide	125-250 μg/mL	125-250 μg/mL	[55]

	Methyl linolenato	3.9-62.5 µg/mL	3.9-250 µg/mL	[55]
	Hexane fraction	3.9-31.2 µg/mL	3.9-31.2 µg/mL	[55]
	Trans-nerolidol	15.6-250 µg/mL	15.6-250 µg/mL	[55]
Annona cornifolia	Squamocin L	150 µg/mL	150 µg/mL	[56]
	Folianin B	75 -150 µg/mL	75 -150 µg/mL	[56]
	Annofolin	9.3 µg/mL	9.3 µg/mL	[56]
	Isolongimicin	18.7 µg/mL	18.7 µg/mL	[56]
	Glaucanisin	75-150 µg/mL	75-150 µg/mL	[56]
	Bullatacin	37.5 µg/mL	37.5 µg/mL	[56]
	Asimicin	150 µg/mL	150 µg/mL	[56]
	Fatty acid methyl esters	0.86-27.7 µg/mL	2-55 µg/mL	[57]
	9-hidroxi-folianina	3.4-27.7 µg/mL	3.4-27.7 µg/mL	[58]
	Sucrose octaacetate	55.5-333 µg/mL	55.5-333 µg/ mL	[58]
Curcuma longa	Curcumin	0.5-32 µg/mL	ND	[59]
Copaifera langsdorffii	Copaiba	62.5 µg/mL	62.5 µg/mL	[60]
**Derivatives Microorganisms**				
Aspergillus felis	Extract	1.9-500 µg/mL	ND	[61]
Alternaria sp.	Extract	500 µg/mL	ND	[61]
Neosartorya sp.	Extract	125-500 µg/mL	ND	[61]
Cladosporium cf. *cladosporioides*	Extract	500 µg/mL	ND	[61]
Hypoxylon cf. *trugodes*	Extract	500 µg/mL	ND	[61]
Neosartorya cf. *udagawae*	Extract	62.5-125 µg/mL	ND	[61]
Eupenicillium javanicum	Extract	500 µg/mL	ND	[61]
Aspergillus lentulus	Extract	500 µg/mL	ND	[61]
Fusarium oxysporum	Extract	500 µg/mL	ND	[61]
Eupenicillium javanicum	Extract	500 µg/mL	ND	[61]
Penicillium crysogenum	Extract	500 µg/mL	ND	[61]
Aspergillus westerdijkiae	Extract	500 µg/mL	ND	[62]
Penicillium cf. *citrinum*	Extract	500 µg/mL	ND	[62]
Aspergillus felis	Cytochalasin E	3.6 µmol	7.2 µM	[63]

Fusarium sp.	Toxin T2	75-640 nmol	75-960 µg/mL	[64]
	8-*n*-butyrylneosolaniol	160-640 nmol	ND	[64]
	8-isobutyrylsolaniol	160-640 nmol	ND	[64]
Alternaria sp.	Altenusin	1.9-31.2 µg/mL	ND	[65]
Galianthe ramosa	Alkaloids (1 and 2)	26-185 µM	ND	[66]
Candida albicans	Farnesol	25 µM	30 µM	[67]

MIC - minimal inhibitory concentration, MFC - minimum fungicidal concentration, ND- not determined

Supplementary Table 3. Antifungal compounds within nanoparticle tested against *Paracoccidioides* spp.

**Compounds**	**Nanoestructured systems**	**MIC**	**MFC**	**Ref.**
Hexyl protocatechuate	Solid Lipid Nanoparticles (cholesterol, phosphate buffer non-ionic surfactant castor oil, polyoxyl-60/PEG-hydrogenated, PS and OS, + Hexyl protocatechuate)	0.24-7.8 µg/mL	0.24-7.8 µg/mL	[68]
Copaifera langsdorffii	Nanoemulsion (Pluronic F-127, ethanol + Copaíba resin oil)	62.5-125 µg/mL	62.5 µg/mL	[69]
Itraconazole	DMSA-PLGA nanoparticles	0.4- 6.25 µg/mL	ND	[70]
Amphotericin B	Magnetite (Fe_3O_4) nanoparticles	0.50 µg/ mL	ND	[71]
Amphotericin B	Poly (lactic-co-glycolic acid) (PLGA) and dimercaptosuccinic acid (DMSA) nanoparticles)	ND	ND	[72,73]

MIC - minimal inhibitory concentration, MFC - minimum fungicidal concentration, ND- not determined

Supplementary Table 4. Patents required to Paracoccidioidomycosis treatment.

Patents required to mycoses treatment				
**Patents applied by universities**				
**Request number**	**Deposit date**	**Title**	**Depositor name**	**Database**
PI0700446-0	02.16.2007	Nanoencapsulated formulation of the drug amphotericin B with dimercaptosuccinic acid in polymer of polylactic-polyglycolic acid for the treatment of mycoses	Universidade de São Paulo	INPI
PI0714221-8	07.13.2007	Compositions and methods for the treatment of mucormycosis and other fungal diseases	Los Angeles Biomedical Research Institute at Har Bor-Ucla Medical Center	INPI / PATENTSCOPE
PI0705676-1	12.18.2007	Use of the plasmidial immunomodulator expressing a mycobacterial stress protein for the control of mycoses	Fundação Universidade de Brasília / Farmacore Biotecnologia Ltda	INPI / PATENTSCOPE
BR102013000829 0	01.11.2013	Pharmaceutical composition for vaginal administration, and, use of the pharmaceutical composition	Glenmark Pharmaceuticals Limited	INPI / PATENTSCOPE
PI1003192-8	02.18.2010	Nanostructured composition the base of P10 immunoprotector peptide based immobilized in polymer blend for the treatment of mycoses	Fundação Universidade de Brasília / Universidade de São Paulo / Universidade Federal de São Paulo	INPI / PATENTSCOPE

PI1101309	03.04.2011	Nanoencapsulated composition of antifungal immobilized in polymeric blend for the treatment of mycoses	Fundação Universidade De Brasília / Universidade De São Paulo	INPI / PATENTSCOPE
CN102283850	08.25.2011	Oil-in-water type compound ketoconazole nano-medicament and preparation method of them	Northwest Agriculture / Forestry University	LATIPAT / ESPACENET / PATENTSCOPE
PI1106432-3	09.23.2011	Aldimine-derived compounds, pharmaceutical compositions and use	Universidade Federal de Minas Gerais / Fundação de Amparo à Pesquisa do Estado de Minas Gerais	INPI / PATENTSCOPE
BR102013032984 3	12.20.2013	Pharmaceutical composition based on the [2-benzenosulfonil-4-(toluene-4-sulfonil)-tiazol-5-yl]-furan-2-ilmethil-amine compound and their use in preparing a drug to infections treatment caused by micro-organisms of the gender *Paracoccidioides* spp.	Fundação Universidade de Brasília / Harmonic Pharma	INPI / PATENTSCOPE
BR102013032983 5	12.20.2013	Pharmaceutical composition based on the 2-[4-alyl-5-(3-chlorine-benzo[b]tiofen-2-yl)-4h-[1,2,4] triazole-3-ilsulfanil]-n-(3-morfolin-4-il-propil)-acetamide compound and their use in the preparation of a drug for the infections treatment caused by micro-organisms of the gender *Paracoccidioides* spp.	Fundação Universidade de Brasília / Harmonic Pharma	INPI / PATENTSCOPE
BR102013032984 3	12.20.2013	Pharmaceutical composition based on the [2-benzenesulfonyl-4-(toluene-4-sulfonyl)-thiazol-5-yl]-furan-2-ilmethyl-amine compound and their use in the preparation of a drug for the infection’s treatment caused by microorganisms of the genus *Paracoccidioides* spp.	Fundação Universidade de Brasília / Harmonic Pharma	INPI / PATENTSCOPE

BR102013032985 1	12.20.2013	Pharmaceutical composition based on 2-[[5-butylsulfanyl-4-(2-phenylethyl)-1,2,4-triazol-3-yl] methylsulfanyl]-4,6-dimethylpyrimidine compound and their use in the preparation of a drug for the infection’s treatment caused by microorganisms of the genus *Paracoccidioides* spp.	Fundação Universidade de Brasília / Harmonic Pharma	INPI / PATENTSCOPE
BR102015001503 8	01.23.2015	Anti-adhesion peptides of *Paracoccidioides* spp. and use of them	Universidade Estadual Paulista Julio de Mesquita Filho	INPI / PATENTSCOPE
BR102016004654 8	03.02.2016	Use of thiosemicarbazide and the thiosemicarbazide camphene derivative in the treatment of mycoses	Universidade Federal de Goiás	INPI / PATENTSCOPE
BR102016021164 6	09.14.2016	Pharmaceutical composition based on 1-chlorine-6-nitro-2-({2-nitrophenyl} sulfanyl)-2,3,3a,4,5,9B-hexahydro-1h-cyclopenta[c]quinoline-4-carboxylic acid compound and their use in the preparation of drugs for the infection’s treatment caused by micro-organisms of the gender *Paracoccidioides* spp.	Universidade Estadual de Maringá	INPI / PATENTSCOPE
BR102017007449 8	04.11.2017	Use of natural compounds in the treatment of mycoses	Universidade Federal de Goiás	INPI / PATENTSCOPE
BR102018009020 8	05.03.2018	Pharmaceutical composition based on 1,3,4-oxadiazolics compounds and their use in the preparation of drugs to systemic infections treat	Universidade Estadual de Maringá / Fundação Universidade de Brasília / Fundação Universidade Do Estado do Mato Grosso	INPI / PATENTSCOPE

BR102018012453 6	06.18.2018	Pharmaceutical composition based on (s)-2-ammonium-3-{(6-bromo-2-methylquinolin-4-yl) thio}propanoate molecule and their use in the preparation of drugs for the infections treatment caused by micro-organisms gender *Paracoccidioides* spp.	Universidade Estadual de Maringá	INPI / PATENTSCOPE
BR102019009329 3	01.17.2019	Use of aryl and heteroaryl chalcone compounds as a growth inhibitor of *Paracoccidioides* spp. in the paracoccidioidomycosis treatment	Universidade Federal de Goiás	INPI
BR102019010517 8	05.23.2019	Antifungal compounds and inhibitors of the enzyme methylcitrate synthase of *Paracoccidioides lutzzi*, promising in the paracoccidioidomycosis treatment	Universidade Federal de Goiás	INPI
BR102019023824 0	11.12.2019	Use of 3-[2,4-Bis((3S)-3-methylmorpholin-4-yl) pyrido [5,6-e]pyrimidin-7-yl]-N-methylbenzamide and 8-(6-Methoxypyridin-3-yl)-3-methyl-1-[4-(piperazin-1-yl)-3-trifluoromethylphenyl]-1,3-dihydroimidazo[4,5-c] quinolin-2-one as a growth inhibitor of *Paracoccidioides* spp. in the paracoccidioidomycosis treatment	Universidade Federal de Goiás	INPI
BR102020014754 4	07.20.2020	Isocitrate lyase inhibitor for the mycosis's treatment	Universidade Federal de Goiás	INPI
BR102020016383 3	08.12.2020	Polymeric nanoformulation of thiosemicarbazide in the mycosis treatment	Universidade Federal de Goiás	INPI
**Patents applied by pharmaceutical industry and other institutions**				
**Request number**	**Deposit date**	**Title**	**Depositor name**	**Database**
PI9707257	01.27.1997	Triazole derivatives useful in therapy	Pfizer Res & Dev	INPI / PATENTSCOPE
02701569	02.20.2002	Water-soluble triazole fungicide	Daiichi Sankyo Co LTD	PATENTSCOPE
03811651	08.01.2003	Dehydrophenylahistins and analogs thereof and the synthesis of dehydrophenylahistins and analogs thereof	Nereus Pharmaceuticals INC.	PATENTSCOPE

05702261	01.13.2005	Combination of voriconazole and an antifungal CYP2C19 inhibitor	Pfizer	INPI / PATENTSCOPE
PI0510417-3	05.27.2005	Particulate-stabilized injectable pharmaceutical compositions of posaconazole	Schering CORP	INPI / PATENTSCOPE
06735282	02.16.2006	Halogen-substituted boronophthalides for the treatment of infections	Anacor Pharmaceuticals INC.	PATENTSCOPE
PI0612103	06.26.2006	Use of a polypeptide or derivative or analogue of the same, and method of preventing and/or treating a fungal contamination and/or by protista	AI2 Limited	INPI / PATENTSCOPE
PI0617739	10.23.2006	Heterocyclic amide derivatives useful as microbicides	Syngenta Participations AG	INPI / PATENTSCOPE
PI0620096	12.19.2006	Alkyl phospholipid derivatives with reduced cytotoxicity and uses of the same	Aeterna Zentaris GMBH	INPI / PATENTSCOPE
PI0712027	05.16.2007	Microbiocidal compounds, method and composition for control and protection against phytopathogenic microorganisms	Syngenta Limited / Syngenta Participations AG	INPI / PATENTSCOPE
PI0712065	06.06.2007	N-(l-alkyl-2-phenyletyl)-carboxamide derivatives and use of these as fungicides	Syngenta Limited / Syngenta Participations AG	INPI / PATENTSCOPE
PI0801803-0	02.08.2008	Combination of substances for the treatment of infectious bacterial, parasitic, fungal and viral diseases understanding the association of an immunomodulator and substances with antibacterial, antiparasitic, antifungal or antiviral action	Iseu da Silva Nunes	INPI / PATENTSCOPE
PI0809238	03.12.2008	Pyridazinone derivatives useful as glycan synthase inhibitors	Albany Molecular Research INC / Schering Corporation	INPI / PATENTSCOPE
09806944	08.07.2009	Antifungal agents	Scynexis INC.	PATENTSCOPE
PI0904249	08.28.2009	Benzylic aralkyl ether compounds, process for preparing the same, intermediate compounds, use of referred compounds, method of treating	Biolab Sanus Farmacêutica LTDA.	INPI / PATENTSCOPE

		and/or preventing, pharmaceutical composition and drugs containing the same		
09793495	12.01.2009	Novel pyrazole-4-N-alkoxycarboxamides as microbiocides	Syngenta Participations AG	INPI / PATENTSCOPE
148254576	12.12.2014	Antimicrobial peptide and uses thereof	Academisch Ziekenhuis Leiden H.O.D.N. LUMC / Academisch Medisch Centrum	PATENTSCOPE

Patents for the treatment of other diseases

Patents applied by universities

**Request number**	**Deposit date**	**Title**	**Depositor name**	**Database**
100114818	08.30.2004	Hydantoin derivatives as inhibitors of cellular necrosis	President and Fellows of Harvard College / The Brigham and Women's Hospital, INC.	PATENTSCOPE
05750392	05.12.2005	Gelsolin for use in treating infections	Brigham and Women's Hospital, INC.	PATENTSCOPE

Patents applied by pharmaceutical industry and other institutions

**Request number**	**Deposit date**	**Title**	**Depositor name**	**Database**
04743172	07.05.2004	Benzimidazole derivatives and their use as protein kinases inhibitors	Astex Therapeutics LTD	PATENTSCOPE

PI0412259-3	07.22.2004	Compound of 1h-pyrazole 3,4-disubstituted and their use as cyclin dependent kinases (CDK) and kinase-3 glycogen synthase (GSK-3) modulators	Astex Therapeutics LTD	INPI / PATENTSCOPE
PI0509150-0	03.24.2005	Compounds for inhibition of angiogenesis and use of these	Jerini AG	INPI / PATENTSCOPE
05716365	03.24.2005	New compounds for the inhibition of angiogenesis and use of thereof	Shire Orphan Therapies GMBH	PATENTSCOPE
PI0512108-6	06.14.2005	Antibiotics containing boronic acid complexes and methods of use	Anacor Pharmaceuticals INC.	INPI / PATENTSCOPE
PI0515316-6	09.14.2005	Imidazoquinoline compounds	Chiron CORP	INPI / PATENTSCOPE
PI0519759-7	12.30.2005	Pharmaceutical compositions	Astex Therapeutics Limited	INPI / PATENTSCOPE
PI0606464-7	01.20.2006	Pharmaceutical compounds, use and process of preparation of the same and pharmaceutical composition	Astex Therapeutics Limited	INPI / PATENTSCOPE
06808472	11.08.2006	Use of pyrroloquinoline compounds to kill clinically latent microorganisms	Helperby Therapeutics LTD	PATENTSCOPE
PI0618374	11.08.2006	Use of a compound or derivative of the same pharmaceutically acceptable, methods to kill clinically latent micro-organisms in a mammalian, to treat or prevent a microbial infection in a mammalian, to sterilize an object, to preserve an inorganic or organic material, to treat a disease, to reduce the conventional antimicrobial agent dose required to treat a microbial infection, and to treat a protozoan disease in a mammalian, combined product, formulation, use of a combined product, compound, and process for preparing the same	Helperby Therapeutics Limited	INPI / PATENTSCOPE
PI0618594	11.13.2006	Microbicides	Syngenta Participations AG	INPI / PATENTSCOPE
PI0706800	01.31.2007	Compounds for the integrins inhibition and use of the same	Jerini AG	INPI / PATENTSCOPE
PI0711659	05.14.2007	Heterocyclic compounds for integrins inhibition and use of the same	Jerini AG	INPI / PATENTSCOPE

07795379	05.25.2007	Triazole compounds that modulate HSP90 activity	Synta Pharmaceuticals CORP	PATENTSCOPE
PI0713010	06.12.2007	Compounds for the treatment of periodontal disease	Anacor Pharmaceuticals INC.	INPI / PATENTSCOPE
PI0716435	08.20.2007	Compounds for the treatment of proliferative diseases	Synta Pharmaceuticals CORP	INPI / PATENTSCOPE
78735222	10.10.2007	Aqueous systems for the preparation of lipid-based pharmaceutical compound, compositions, methods, and uses thereof	Jina Pharmaceuticals INC.	PATENTSCOPE
121876114	10.12.2007	Hydrobenzamide derivatives as inhibitors of HSP90	Astex Therapeutics Limited	PATENTSCOPE
87506267	05.16.2008	Use of 4-(pyrrolidin-1-yl) quinoline compounds to kill clinically latent microorganisms	Helperby Therapeutics Limited	PATENTSCOPE
88266085	07.28.2008	Antimicrobial peptide and compositions thereof	Revance Therapeutics INC.	PATENTSCOPE
88756580	10.24.2008	Use of alginate oligomers in combating biofilms	Algipharma AS	PATENTSCOPE
97182984	02.27.2009	Compounds and compositions as TLR activity modulators	Novartis AG	PATENTSCOPE
09731468	04.09.2009	Pharmaceutical compounds	Astex Therapeutics LTD	PATENTSCOPE
117143792	03.29.2011	Novel combination and use	Helperby Therapeutics Limited	PATENTSCOPE
117582577	09.09.2011	Combination of phenoxybenzamine and polymixine e to treat microbial infections	Helperby Therapeutics Limited	PATENTSCOPE
128581907	12.10.2012	Metalloenzyme inhibitor compounds	Viamet Pharmaceuticals (NC), INC.	PATENTSCOPE
147276497	03.13.2014	Macrocyclic inhibitors of the PD-1/PD-L1 and CD80(B7-1)/PD-L1 protein/protein interactions	Bristol-Myers Squibb Company	INP / PATENTSCOPE
148428220	09.05.2014	1,3,4-oxadiazole and 1,3,4-thiadiazole derivatives as immunomodulators	Aurigene Discovery Technologies Limited	INPI / PATENTSCOPE
147819478	09.05.2014	Cyclic peptidomimetic compounds as immunomodulators	Aurigene Discovery Technologies Limited	PATENTSCOPE

147903207	09.05.2014	1,2,4-oxadiazole derivatives as immunomodulators	Aurigene Discovery Technologies Limited	INPI / PATENTSCOPE
BR112017012704 0	12.17.2015	Immunomodulators	Bristol-Myers Squibb Company	INPI / PATENTSCOPE

References

1. Hahn, R.C.; Hamdan, J.S. Effects of amphotericin B and three azole derivatives on the Lipids of yeast cells of Paracoccidioides brasiliensis. *Antimicrobial Agents and Chemotherapy* **2000**, *44*, 1997–2000, doi:10.1128/AAC.44.7.1997-2000.2000.

2. Cruz, R.C.; Werneck, S.M.C.; Oliveira, C.S.; Santos, P.C.; Soares, B.M.; Santos, D.A.; Cisalpino, P.S. Influence of Different Media, Incubation Times, and Temperatures for Determining the MICs of Seven Antifungal Agents against Paracoccidioides brasiliensis by Microdilution. *Journal of Clinical Microbiology* **2013**, *51*, 436–443, doi:10.1128/JCM.02231-12.

3. Takahagi-Nakaira, E.; Sugizaki, M.F.; Peraçoli, M.T.S. Microdilution procedure for antifungal susceptibility testing of Paracoccidioides brasiliensis to amphotericin B and itraconazole. *Journal of Venomous Animals and Toxins including Tropical Diseases* **2009**, *15*, 718–731.

4. Restrepo, A.; Tabares, C. de B.A.M. In vitro susceptibility of Paracoccidioides brasiliensis yeast form to antifungal agents. *Revista do Instituto de Medicina Tropical de São Paulo* **1984**, *26*, 322–328, doi:10.1590/S0036-46651984000600006.

5. de Oliveira, A.A.; Neves, B.J.; Silva, L. do C.; Soares, C.M. de A.; Andrade, C.H.; Pereira, M. Drug Repurposing for Paracoccidioidomycosis Through a Computational Chemogenomics Framework. *Frontiers in Microbiology* **2019**, *10*, 1301, doi:10.3389/fmicb.2019.01301.

6. Hahn, R.C.; Fontes, C.J.F.; Batista, R.D.; Hamdan, J.S. In Vitro comparison of activities of Terbinafine and Itraconazole against Paracoccidioides brasiliensis. *Journal of Clinical Microbiology* **2002**, *40*, 2828–2831, doi:10.1128/JCM.40.8.2828-2831.2002.

7. San-Blas, G.; Calcagno, A.M.; San-Blas, F. A preliminary study of in vitro antibiotic activity of saperconazole and other azoles on Paracoccidioides brasiliensis. *J. Med. Vet. Mycol.* **1993**, *31*, 169–174.

8. Lino, C.I.; Gonçalves de Souza, I.; Borelli, B.M.; Silvério Matos, T.T.; Santos Teixeira, I.N.; Ramos, J.P.; Maria de Souza Fagundes, E.; de Oliveira Fernandes, P.; Maltarollo, V.G.; Johann, S.; et al. Synthesis, molecular modeling studies and evaluation of antifungal activity of a novel series of thiazole derivatives. *European Journal of Medicinal Chemistry* **2018**, *151*, 248–260, doi:10.1016/j.ejmech.2018.03.083.

9. Rodríguez-Brito, S.; Niño-Vega, G.; San-Blas, G. Caspofungin Affects Growth of Paracoccidioides brasiliensis in Both Morphological Phases. *Antimicrobial Agents and Chemotherapy* **2010**, *54*, 5391–5394, doi:10.1128/AAC.00617-10.

10. Nakai, T.; Uno, J.; Ikeda, F.; Tawara, S.; Nishimura, K.; Miyaji, M. In Vitro Antifungal Activity of Micafungin (FK463) against Dimorphic Fungi: Comparison of Yeast-Like and Mycelial Forms. *Antimicrobial Agents and Chemotherapy* **2003**, *47*, 1376–1381, doi:10.1128/AAC.47.4.1376-1381.2003.

11. Restrepo, A.; Arango, M.D. In vitro susceptibility testing of Paracoccidioides brasiliensis to sulfonamides. *Antimicrobial Agents and Chemotherapy* **1980**, *18*, 190–194,

doi:10.1128/AAC.18.1.190.

12. Thomaz, L.; Apitz-Castro, R.; Marques, A.F.; Travassos, L.R.; Taborda, C.P. Experimental paracoccidioidomycosis: alternative therapy with ajoene, compound from Allium sativum , associated with sulfamethoxazole/trimethoprim. *Medical Mycology* **2008**, *46*, 113–118, doi:10.1080/13693780701651681.

13. Souza, M.A.; Johann, S.; Lima, L.A.R. dos S.; Campos, F.F.; Mendes, I.C.; Beraldo, H.; Souza-Fagundes, E.M. de; Cisalpino, P.S.; Rosa, C.A.; Alves, T.M. de A. The antimicrobial activity of lapachol and its thiosemicarbazone and semicarbazone derivatives. *Memórias do Instituto Oswaldo Cruz* **2013**, *108*, 342–351.

14. de Sá, N.P.; Cisalpino, P.S.; Bertollo, C.M.; Santos, P.C.; Rosa, C.A.; de Souza, D. da G.; Barbeira, P.J.S.; Alves, T.M. de A.; Zani, C.L.; Johann, S. Thiosemicarbazone of lapachol acts on cell membrane in Paracoccidioides brasiliensis. *Medical Mycology* **2019**, *57*, 332–339, doi:10.1093/mmy/myy045.

15. Borba, J.V.V.B.; Tauhata, S.B.F.; Oliveira, C.M.A. de; Ferreira Marques, M.; Bailão, A.M.; Soares, C.M. de A.; Pereira, M. Chemoproteomic identification of molecular targets of antifungal prototypes, thiosemicarbazide and a camphene derivative of thiosemicarbazide, in Paracoccidioides brasiliensis. *PLOS ONE* **2018**, *13*, e0201948, doi:10.1371/journal.pone.0201948.

16. do Carmo Silva, L.; Tamayo Ossa, D.P.; Castro, S.V.D.C.; Bringel Pires, L.; Alves de Oliveira, C.M.; Conceição da Silva, C.; Coelho, N.P.; Bailão, A.M.; Parente-Rocha, J.A.; Soares, C.M.D.A.; et al. Transcriptome Profile of the Response of Paracoccidioides spp. to a Camphene Thiosemicarbazide Derivative. *PLOS ONE* **2015**, *10*, e0130703, doi:10.1371/journal.pone.0130703.

17. de Sá, N.P.; Cisalpino, P.S.; Tavares, L.C.; Espíndola, L.; Borelli, B.M.; Barbeira, P.J.; Cardoso Perdigão, G. de M.; Souza-Fagundes, E.M.; Rosa, C.A.; Pizzolatti, M.G.; et al. Effects of two 6-quinolinyl chalcones on the integrity of plasma membrane of Paracoccidioides brasiliensis. *The Journal of Antibiotics* **2017**, *70*, 277–284, doi:10.1038/ja.2016.156.

18. de Carvalho Tavares, L.; Johann, S.; Maria de Almeida Alves, T.; Guerra, J.C.; Maria de Souza-Fagundes, E.; Cisalpino, P.S.; Bortoluzzi, A.J.; Caramori, G.F.; de Mattos Piccoli, R.; Braibante, H.T.S.; et al. Quinolinyl and quinolinyl N-oxide chalcones: Synthesis, antifungal and cytotoxic activities. *European Journal of Medicinal Chemistry* **2011**, *46*, 4448–4456, doi:10.1016/j.ejmech.2011.07.019.

19. de Sá, N.P.; Cisalpino, P.S.; Tavares, L. de C.; Espíndola, L.; Pizzolatti, M.G.; Santos, P.C.; de Paula, T.P.; Rosa, C.A.; de Souza, D. da G.; Santos, D.A.; et al. Antifungal activity of 6-quinolinyl N-oxide chalcones against Paracoccidioides. *Journal of Antimicrobial Chemotherapy* **2015**, *70*, 841–845, doi:10.1093/jac/dku427.

20. Silva, L.C.; Neves, B.J.; Gomes, M.N.; Melo-Filho, C.C.; Soares, C.M.; Andrade, C.H.; Pereira, M. Computer-aided identification of novel anti-paracoccidioidomycosis compounds. *Future Microbiol* **2018**, *13*, 1523–1535, doi:10.2217/fmb-2018-0175.

21. Horwath, M.C.; Bell-Horwath, T.R.; Lescano, V.; Krishnan, K.; Merino, E.J.; Deepe,

G.S. Antifungal Activity of the Lipophilic Antioxidant Ferrostatin-1. *ChemBioChem* **2017**, *18*, 2069–2078, doi:10.1002/cbic.201700105.

22. de Paula e Silva, A.C.A.; Costa-Orlandi, C.B.; Gullo, F.P.; Sangalli-Leite, F.; de Oliveira, H.C.; Silva, J. de F. da; Scorzoni, L.; Pitangui, N. de S.; Rossi, S.A.; Benaducci, T.; et al. Antifungal Activity of Decyl Gallate against Several Species of Pathogenic Fungi. *Evidence-Based Complementary and Alternative Medicine* **2014**, *2014*, 1–8, doi:10.1155/2014/506273.

23. De Paula E Silva, A.C.A.; De Oliveira, H.C.; Scorzoni, L.; Marcos, C.M.; Dos Santos, C.T.; Fusco-Almeida, A.M.; Guerta Salina, A.C.; Medeiros, A.I.; Almeida, F.; Li, S.C.; et al. Decyl gallate as a possible inhibitor of N-glycosylation process in paracoccidioides lutzii. *Antimicrobial Agents and Chemotherapy* **2019**, *63*, doi:10.1128/AAC.01909-18.

24. Donnici, C.L.; Nogueira, L.J.; Araujo, M.H.; Oliveira, S.R.; Magalhães, T.F.F.; Lopes, M.T.P.; e Silva, A.C.; Ferreira, A.M. da C.; Martins, C.V.B.; de Resende Stoianoff, M.A. In vitro studies of the activity of dithiocarbamate organoruthenium complexes against clinically relevant fungal pathogens. *Molecules* **2014**, *19*, 5402–5420, doi:10.3390/molecules19045402.

25. Hanson, L.H.; Stevens, D.A. Evaluation of cilofungin, a lipopeptide antifungal agent, in vitro against fungi isolated from clinical specimens. *Antimicrob. Agents Chemother.* **1989**, *33*, 1391–1392, doi:10.1128/aac.33.8.1391.

26. Arruda, D.C.; Matsuo, A.L.; Silva, L.S.; Real, F.; Leitão, N.P.; Pires, J.H.S.; Caires, A.C.F.; Garcia, D.M.; Cunha, F.F.M.; Puccia, R.; et al. Cyclopalladated Compound 7a Induces Apoptosis- and Autophagy-Like Mechanisms in Paracoccidioides and Is a Candidate for Paracoccidioidomycosis Treatment. *Antimicrobial Agents and Chemotherapy* **2015**, *59*, 7214–7223, doi:10.1128/AAC.00512-15.

27. Rodrigues-Vendramini, F.A.V.; Marschalk, C.; Toplak, M.; Macheroux, P.; Bonfim-Mendonça, P. de S.; Svidzinski, T.I.E.; Seixas, F.A.V.; Kioshima, E.S. Promising New Antifungal Treatment Targeting Chorismate Synthase from Paracoccidioides brasiliensis. *Antimicrobial Agents and Chemotherapy* **2018**, *63*, doi:10.1128/AAC.01097-18.

28. Bueno, P.S.A.; Rodrigues-Vendramini, F.A.V.; Toplak, M.; Macheroux, P.; Kioshima, É.S.; Seixas, F.A.V. New inhibitors of chorismate synthase present antifungal activity against Paracoccidioides brasiliensis. *Future Microbiology* **2019**, *14*, 969–980, doi:10.2217/fmb-2019-0052.

29. Da Silva, L.S.; Barbosa, U.R.; Silva, L.D.C.; Soares, C.M.A.; Pereira, M.; Da Silva, R.A. Identification of a new antifungal compound against isocitrate lyase of Paracoccidioides brasiliensis. *Future Microbiology* **2020**, *14*, 1589–1606, doi:10.2217/fmb-2019-0166.

30. Prado, R.S. do; Alves, R.J.; Oliveira, C.M.A. de; Kato, L.; Silva, R.A. da; Quintino, G.O.; do Desterro Cunha, S.; de Almeida Soares, C.M.; Pereira, M. Inhibition of Paracoccidioides lutzii Pb01 Isocitrate Lyase by the Natural Compound Argentilactone and Its Semi-Synthetic Derivatives. *PLoS ONE* **2014**, *9*, e94832, doi:10.1371/journal.pone.0094832.

31. Rossi, D.C.P.; Spadari, C. de C.; Nosanchuk, J.D.; Taborda, C.P.; Ishida, K. Miltefosine is fungicidal to Paracoccidioides spp. yeast cells but subinhibitory concentrations induce melanisation. *International Journal of Antimicrobial Agents* **2017**, *49*, 465–471,

doi:10.1016/j.ijantimicag.2016.12.020.

32. Rodrigues-Vendramini, F.A.V.; Faria, D.R.; Arita, G.S.; Capoci, I.R.G.; Sakita, K.M.; Caparroz-Assef, S.M.; Becker, T.C.A.; de Souza Bonfim-Mendonça, P.; Felipe, M.S.; Svidzinski, T.I.E.; et al. Antifungal activity of two oxadiazole compounds for the paracoccidioidomycosis treatment. *PLOS Neglected Tropical Diseases* **2019**, *13*, e0007441, doi:10.1371/journal.pntd.0007441.

33. Abadio, A.K.R.; Kioshima, E.S.; Leroux, V.; Martins, N.F.; Maigret, B.; Felipe, M.S.S. Identification of new antifungal compounds targeting thioredoxin reductase of Paracoccidioides genus. *PLoS ONE* **2015**, *10*, doi:10.1371/journal.pone.0142926.

34. Capoci, I.R.G.; Faria, D.R.; Sakita, K.M.; Rodrigues-Vendramini, F.A.V.; Bonfim-Mendonça, P. de S.; Becker, T.C.A.; Kioshima, É.S.; Svidzinski, T.I.E.; Maigret, B. Repurposing approach identifies new treatment options for invasive fungal disease. *Bioorganic Chemistry* **2019**, *84*, 87–97, doi:10.1016/j.bioorg.2018.11.019.

35. Bagatin, M.C.; Pimentel, A.L.; Biavatti, D.C.; Basso, E.A.; Kioshima, E.S.; Seixas, F.A. V; Gauze, G. de F. Targeting the Homoserine Dehydrogenase of Paracoccidioides Species for Treatment of Systemic Fungal Infections. *Antimicrobial Agents and Chemotherapy* **2017**, *61*, doi:10.1128/AAC.00165-17.

36. Bueno, P.S.A.; Rodrigues, F.A.V.; Santos, J.L.; Canduri, F.; Biavatti, D.C.; Pimentel, A.L.; Bagatin, M.C.; Kioshima, É.S.; de Freitas Gauze, G.; Seixas, F.A.V. New inhibitors of homoserine dehydrogenase from Paracoccidioides brasiliensis presenting antifungal activity. *Journal of Molecular Modeling* **2019**, *25*, doi:10.1007/s00894-019-4221-2.

37. Bagatin, M.C.; F Rozada, A.M.; V Rodrigues, F.A.; A Bueno, P.S.; Santos, J.L.; Canduri, F.; Kioshima, É.S.; V Seixas, F.A.; Basso, E.A.; Gauze, G.F. New 4-methoxy-naphthalene derivatives as promisor antifungal agents for paracoccidioidomycosis treatment. *Future Microbiol* **2019**, *14*, 235–245, doi:10.2217/fmb-2018-0276.

38. Rozada, A.M.F.; Rodrigues, F.A.V.; Sampiron, E.G.; Seixas, F.A.V.; Basso, E.A.; Scodro, R.B.L.; Kioshima, É.S.; Gauze, G.F. Novel 4-methoxynaphthalene-N-acylhydrazones as potential agents for the treatment of paracoccidioidomycosis and tuberculosis co-infection. *Future Microbiology* **2019**, *14*, 587–598, doi:10.2217/fmb-2018-0357.

39. Visbal, G.; Alvarez, A.; Moreno, B.; San-Blas, G. S-adenosyl-L-methionine inhibitors Δ24-sterol methyltransferase and Δ24 (28)-sterol methylreductase as possible agents against Paracoccidioides brasiliensis. *Antimicrobial agents and chemotherapy* **2003**, *47*, 2966–2970.

40. Visbal, G.; San-Blas, G.; Maldonado, A.; Álvarez-Aular, Á.; Capparelli, M. V.; Murgich, J. Synthesis, in vitro antifungal activity and mechanism of action of four sterol hydrazone analogues against the dimorphic fungus Paracoccidioides brasiliensis. *Steroids* **2011**, *76*, 1069–1081, doi:10.1016/j.steroids.2011.04.012.

41. Costa, F.G.; Neto, B.R. da S.; Gonçalves, R.L.; da Silva, R.A.; de Oliveira, C.M.A.; Kato, L.; Freitas, C.D.S.; Giannini, M.J.S.M.; da Silva, J. de F.; Soares, C.M. de A.; et al. Alkaloids as Inhibitors of Malate Synthase from Paracoccidioides spp.: Receptor-Ligand Interaction-Based Virtual Screening and Molecular Docking Studies, Antifungal Activity, and the Adhesion Process. *Antimicrobial Agents and Chemotherapy* **2015**, *59*, 5581–5594,

doi:10.1128/AAC.04711-14.

42. Singulani, J. de L.; Galeane, M.C.; Ramos, M.D.; Gomes, P.C.; dos Santos, C.T.; de Souza, B.M.; Palma, M.S.; Fusco Almeida, A.M.; Mendes Giannini, M.J.S. Antifungal Activity, Toxicity, and Membranolytic Action of a Mastoparan Analog Peptide. *Frontiers in Cellular and Infection Microbiology* **2019**, *9*, 419, doi:10.3389/fcimb.2019.00419.

43. Fernandes, K.E.; Carter, D.A. The Antifungal Activity of Lactoferrin and Its Derived Peptides: Mechanisms of Action and Synergy with Drugs against Fungal Pathogens. *Frontiers in Microbiology* **2017**, *8*, 2, doi:10.3389/fmicb.2017.00002.

44. Coimbra de Oliveira, M.; Souza Reis, F.; de Fatima, A.; Furtado Ferreira Magalhaes, T.; Leticia da Silva, D.; Rodrigues Porto, R.; Almeida Watanabe, G.; Viviane Buzanello Martins, C.; Leite da Silva, D.; Lucia Tasca Gois Ruiz, A.; et al. Synthesis and Anti-Paracoccidioides Activity of Calix[n]arenes. *Letters in Drug Design & Discovery* **2012**, *9*, 30–36, doi:10.2174/157018012798192991.

45. de Oliveira, A.A.; Neves, B.J.; Silva, L. do C.; Soares, C.M. de A.; Andrade, C.H.; Pereira, M. Drug Repurposing for Paracoccidioidomycosis Through a Computational Chemogenomics Framework. *Frontiers in Microbiology* **2019**, *10*, 1301, doi:10.3389/fmicb.2019.01301.

46. Silva, L. do C.; Tauhata, S.B.F.; Baeza, L.C.; de Oliveira, C.M.A.; Kato, L.; Borges, C.L.; de Almeida Soares, C.M.; Pereira, M. Argentilactone Molecular Targets in Paracoccidioides brasiliensis Identified by Chemoproteomics. *Antimicrob. Agents Chemother.* **2018**, *62*, doi:10.1128/AAC.00737-18.

47. Santos, G.D.; Ferri, P.H.; Santos, S.C.; Bao, S.N.; Soares, C.M.A.; Pereira, M. Oenothein B inhibits the expression of PbFKS1 transcript and induces morphological changes in Paracoccidioides brasiliensis. *Medical Mycology* **2007**, *45*, 609–618, doi:10.1080/13693780701502108.

48. Costa, D.P.; Filho, E.G.A.; Silva, L.M.A.; Santos, S.C.; Passos, X.S.; Do Rosário R. Silva, M.; Seraphin, J.C.; Ferri, P.H. Influence of fruit biotypes on the chemical composition and antifungal activity of the essential oils of eugenia uniflora leaves. *Journal of the Brazilian Chemical Society* **2010**, *21*, 851–858, doi:10.1590/S0103-50532010000500012.

49. Johann, S.; Sá, N.P.; Lima, L.A.R.S.; Cisalpino, P.S.; Cota, B.B.; Alves, T.M.A.; Siqueira, E.P.; Zani, C.L. Antifungal activity of schinol and a new biphenyl compound isolated from Schinus terebinthifolius against the pathogenic fungus Paracoccidioides brasiliensis. *Annals of Clinical Microbiology and Antimicrobials* **2010**, *9*, 30, doi:10.1186/1476-0711-9-30.

50. Furtado, F.; Borges, B.; Teixeira, T.; Garces, H.; Almeida Junior, L.; Alves, F.; Silva, C.; Fernandes Junior, A. Chemical Composition and Bioactivity of Essential Oil from Blepharocalyx salicifolius. *International Journal of Molecular Sciences* **2018**, *19*, 33, doi:10.3390/ijms19010033.

51. Ruas, L.P.; Carvalho, F.C.; Roque-Barreira, M.C. ArtinM offers new perspectives in the development of antifungal therapy. *Frontiers in Microbiology* **2012**, *3*, doi:10.3389/fmicb.2012.00218.

52. Thomaz, L.; Apitz-Castro, R.; Marques, A.F.; Travassos, L.R.; Taborda, C.P. Experimental paracoccidioidomycosis: alternative therapy with ajoene, compound from Allium sativum , associated with sulfamethoxazole/trimethoprim. *Medical Mycology* **2008**, *46*, 113–118, doi:10.1080/13693780701651681.

53. do Prado, A.C.; Garces, H.G.; Bagagli, E.; Rall, V.L.M.; Furlanetto, A.; Fernandes Junior, A.; Furtado, F.B. Schinus molle essential oil as a potential source of bioactive compounds: antifungal and antibacterial properties. *J. Appl. Microbiol.* **2019**, *126*, 516–522, doi:10.1111/jam.14157.

54. Pinto, M.E.A.; Araújo, S.G.; Morais, M.I.; Sá, N.P.; Lima, C.M.; Rosa, C.A.; Siqueira, E.P.; Johann, S.; Lima, L.A.R.S. Antifungal and antioxidant activity of fatty acid methyl esters from vegetable oils. *Anais da Academia Brasileira de Ciencias* **2017**, *89*, 1671–1681, doi:10.1590/0001-3765201720160908.

55. Johann, S.; Cisalpino, P.S.; Watanabe, G.A.; Cota, B.B.; de Siqueira, E.P.; Pizzolatti, M.G.; Zani, C.L.; de Resende, M.A. Antifungal activity of extracts of some plants used in Brazilian traditional medicine against the pathogenic fungus Paracoccidioides brasiliensis. *Pharmaceutical Biology* **2010**, *48*, 388–396, doi:10.3109/13880200903150385.

56. Johann, S.; Oliveira, F.B.; Siqueira, E.P.; Cisalpino, P.S.; Rosa, C.A.; Alves, T.M.A.; Zani, C.L.; Cota, B.B. Activity of compounds isolated from Baccharis dracunculifolia D.C. (Asteraceae) against Paracoccidioides brasiliensis. *Medical Mycology* **2012**, *50*, 843–851, doi:10.3109/13693786.2012.678903.

57. LIMA, L.A.R.S.; ALVES, T.M.A.; ZANI, C.L.; SALES JÚNIOR, P.A.; ROMANHA, A.J.; JOHANN, S.; CISALPINO, P.S.; PIMENTA, L.P.S.; BOAVENTURA, M.A.D. In vitro cytotoxic, antifungal, trypanocidal and leishmanicidal activities of acetogenins isolated from Annona cornifolia A. St. -Hil. (Annonaceae). *Anais da Academia Brasileira de Ciências* **2014**, *86*, 829–839, doi:10.1590/0001-3765201420130048.

58. Lima, L.A.R. dos S.; Johann, S.; Cisalpino, P.S.; Pimenta, L.P.S.; Boaventura, M.A.D. In vitro antifungal activity of fatty acid methyl esters of the seeds of Annona cornifolia A.St.-Hil. (Annonaceae) against pathogenic fungus Paracoccidioides brasiliensis. *Revista da Sociedade Brasileira de Medicina Tropical* **2011**, *44*, 777–780, doi:10.1590/S0037-86822011000600024.

59. Lima, L.A.R.S.; Johann, S.; Cisalpino, P.S.; Pimenta, L.P.S.; Boaventura, M.A.D. Antifungal activity of 9-hydroxy-folianin and sucrose octaacetate from the seeds of Annona cornifolia A. St. -Hil. (Annonaceae). *Food Research International* **2011**, *44*, 2283–2288, doi:10.1016/j.foodres.2010.11.030.

60. Martins, C.V.B.; da Silva, D.L.; Neres, A.T.M.; Magalhães, T.F.F.; Watanabe, G.A.; Modolo, L. V; Sabino, A.A.; de Fátima, A.; de Resende, M.A. Curcumin as a promising antifungal of clinical interest. *J. Antimicrob. Chemother.* **2009**, *63*, 337–339, doi:10.1093/jac/dkn488.

61. do Carmo Silva, L.; Miranda, M.A.C.M.; de Freitas, J.V.; Ferreira, S.F.A.; de Oliveira Lima, E.C.; de Oliveira, C.M.A.; Kato, L.; Terezan, A.P.; Rodriguez, A.F.R.; Faria, F.S.E.D.V.; et al. Antifungal activity of Copaíba resin oil in solution and nanoemulsion against Paracoccidioides spp. *Braz J Microbiol* **2020**, *51*, 125–134, doi:10.1007/s42770-019-

00201-3.

62. Mendes, G.; Gonçalves, V.N.; Souza-Fagundes, E.M.; Kohlhoff, M.; Rosa, C.A.; Zani, C.L.; Cota, B.B.; Rosa, L.H.; Johann, S. Antifungal activity of extracts from Atacama Desert fungi againstParacoccidioides brasiliensis and identification ofAspergillus felis as a promising source of natural bioactive compounds. *Memórias do Instituto Oswaldo Cruz* **2016**, *111*, 209–217, doi:10.1590/0074-02760150451.

63. Mendes, G.; Baltazar, L.M.; Souza, D.G.; Sá, N.P.; Rosa, L.H.; Rosa, C.A.; Souza-Fagundes, E.M.; Ramos, J.P.; Alves-Silva, J.; Cota, B.B.; et al. Effects of cytochalasin E on Paracoccidioides brasiliensis. *Journal of Applied Microbiology* **2018**, *125*, 1296–1307, doi:10.1111/jam.14053.

64. Campos, F.F.; Johann, S.; Cota, B.B.; Alves, T.M.A.; Rosa, L.H.; Caligiorne, R.B.; Cisalpino, P.S.; Rosa, C.A.; Zani, C.L. Antifungal activity of trichothecenes from Fusarium sp. against clinical isolates of Paracoccidioides brasiliensis. *Mycoses* **2011**, *54*, doi:10.1111/j.1439-0507.2009.01854.x.

65. Johann, S.; Rosa, L.H.; Rosa, C.A.; Perez, P.; Cisalpino, P.S.; Zani, C.L.; Cota, B.B. Antifungal activity of altenusin isolated from the endophytic fungus Alternaria sp. against the pathogenic fungus Paracoccidioides brasiliensis. *Revista Iberoamericana de Micología* **2012**, *29*, 205–209, doi:10.1016/j.riam.2012.02.002.

66. De Freitas, C.S.; Kato, L.; De Oliveira, C.M.A.; Queiroz, L.H.K.; Santana, M.J.; Schuquel, I.T.; Delprete, P.G.; Da Silva, R.A.; Quintino, G.O.; Da Silva Neto, B.R.; et al. β-carboline alkaloids from Galianthe ramosa inhibit malate synthase from Paracoccidioides spp. *Planta Medica* **2014**, *80*, 1746–1752, doi:10.1055/s-0034-1383305.

67. Derengowski, L.S.; De-Souza-Silva, C.; Braz, S. V; Mello-De-Sousa, T.M.; Báo, S.N.; Kyaw, C.M.; Silva-Pereira, I. Antimicrobial effect of farnesol, a Candida albicans quorum sensing molecule, on Paracoccidioides brasiliensis growth and morphogenesis. *Annals of Clinical Microbiology and Antimicrobials* **2009**, *8*, 13, doi:10.1186/1476-0711-8-13.

68. Medina-Alarcón, K.P.; Singulani, J.L.; Voltan, A.R.; Sardi, J.C.O.; Petrônio, M.S.; Santos, M.B.; Polaquini, C.R.; Regasini, L.O.; Bolzani, V.S.; da Silva, D.H.S.; et al. Alkyl Protocatechuate-Loaded Nanostructured Lipid Systems as a Treatment Strategy for Paracoccidioides brasiliensis and Paracoccidioides lutzii In Vitro. *Frontiers in Microbiology* **2017**, *8*, 1048, doi:10.3389/fmicb.2017.01048.

69. do Carmo Silva, L.; Miranda, M.A.C.M.; de Freitas, J.V.; Ferreira, S.F.A.; de Oliveira Lima, E.C.; de Oliveira, C.M.A.; Kato, L.; Terezan, A.P.; Rodriguez, A.F.R.; Faria, F.S.E.D.V.; et al. Antifungal activity of Copaíba resin oil in solution and nanoemulsion against Paracoccidioides spp. *Brazilian Journal of Microbiology* **2020**, *51*, 125–134, doi:10.1007/s42770-019-00201-3.

70. Cunha-Azevedo, E.P.; Silva, J.R.; Martins, O.P.; Siqueira-Moura, M.P.; Bocca, A.L.; Felipe, M.S.S.; Tedesco, A.C.; Azevedo, R.B. In vitro antifungal activity and toxicity of itraconazole in DMSA-PLGA nanoparticles. In Proceedings of the Journal of Nanoscience and Nanotechnology; 2011; Vol. 11, pp. 2308–2314.

71. Saldanha, C.A.; Garcia, M.P.; Iocca, D.C.; Rebelo, L.G.; Souza, A.C.O.; Bocca, A.L.;

Almeida Santos, M. de F.M.; Morais, P.C.; Azevedo, R.B. Antifungal Activity of Amphotericin B Conjugated to Nanosized Magnetite in the Treatment of Paracoccidioidomycosis. *PLOS Neglected Tropical Diseases* **2016**, *10*, e0004754, doi:10.1371/journal.pntd.0004754.

72. Souza, A.C.O.; Nascimento, A.L.; de Vasconcelos, N.M.; Jerônimo, M.S.; Siqueira, I.M.; R-Santos, L.; Cintra, D.O.S.; Fuscaldi, L.L.; Pires Júnior, O.R.; Titze-de-Almeida, R.; et al. Activity and in vivo tracking of Amphotericin B loaded PLGA nanoparticles. *European Journal of Medicinal Chemistry* **2015**, *95*, 267–276, doi:10.1016/j.ejmech.2015.03.022.

73. Amaral, A.C.; Bocca, A.L.; Ribeiro, A.M.; Nunes, J.; Peixoto, D.L.G.; Simioni, A.R.; Primo, F.L.; Lacava, Z.G.M.; Bentes, R.; Titze-de-Almeida, R.; et al. Amphotericin B in poly(lactic-co-glycolic acid) (PLGA) and dimercaptosuccinic acid (DMSA) nanoparticles against paracoccidioidomycosis. *Journal of Antimicrobial Chemotherapy* **2009**, *63*, 526–533, doi:10.1093/jac/dkn539.
